# Determinants of Acquisition, Persistence, and Clearance of Oncogenic Cervical Human Papillomavirus Infection in the Philippines Using a Multi-Omics Approach: DEFEAT HPV Study Protocol

**DOI:** 10.3390/healthcare11050658

**Published:** 2023-02-23

**Authors:** Sheriah Laine M. de Paz-Silava, Ian Kim B. Tabios, Ourlad Alzeus G. Tantengco, Fresthel Monica M. Climacosa, Clarissa L. Velayo, Ryan C. V. Lintao, Leslie Faye T. Cando, Glenmarie Angelica S. Perias, Maria Isabel C. Idolor, Abialbon G. Francisco, Charlene Divine M. Catral, Charlotte M. Chiong, Leslie Michele M. Dalmacio

**Affiliations:** 1 Multi-Omics Research Program for Health, College of Medicine, University of the Philippines Manila, Manila 1000, Philippines; 2 Department of Medical Microbiology, College of Public Health, University of the Philippines Manila, Manila 1000, Philippines; 3 Department of Biochemistry and Molecular Biology, College of Medicine, University of the Philippines Manila, Manila 1000, Philippines; 4 Institute of Biology, College of Science, University of the Philippines Diliman, Quezon City 1101, Philippines; 5 Department of Physiology, College of Medicine, University of the Philippines Manila, Manila 1000, Philippines; 6 Department of Biology, College of Science, De La Salle University, Manila 0922, Philippines; 7 Division of Maternal-Fetal Medicine, Department of Obstetrics and Gynecology, University of the Philippines College of Medicine—Philippine General Hospital, University of the Philippines Manila, Manila 1000, Philippines; 8 Department of Otolaryngology—Head and Neck Surgery, University of the Philippines College of Medicine—Philippine General Hospital, Manila 1000, Philippines

**Keywords:** human papillomavirus, cervical cancer, epidemiology, vaccination, screening, treatment, Philippines

## Abstract

HPV infection is one of the most studied risk factors in cervical cancer—the second most common cancer site and cause of death due to cancer in the Philippines. However, there is a lack of population-based epidemiological data on cervical HPV infection in the Philippines. Local reports on co-infections with other lower genital tract pathogens, commonly reported globally, are also lacking, which emphasizes the need to increase efforts in targeting HPV prevalence, genotype, and distribution. Hence, we aim to determine the molecular epidemiology and natural history of HPV infection among reproductive-age Filipino women using a community-based prospective cohort design. Women from rural and urban centers will be screened until the target sample size of 110 HPV-positive women (55 from rural sites and 55 from urban sites) is reached. Cervical and vaginal swabs will be collected from all screened participants. For HPV-positive patients, HPV genotypes will be determined. One hundred ten healthy controls will be selected from previously screened volunteers. The cases and controls will comprise the multi-omics subset of participants and will be followed up after 6 and 12 months for repeat HPV screening. Metagenomic and metabolomic analyses of the vaginal swabs will also be performed at baseline, after 6 months, and after 12 months. The results of this study will update the prevalence and genotypic distribution of cervical HPV infection among Filipino women, determine whether the current vaccines used for HPV vaccination programs capture the most prevalent high-risk HPV genotypes in the country, and identify vaginal community state types and bacterial taxa associated with the natural history of cervical HPV infection. The results of this study will be used as the basis for developing a biomarker that can help predict the risk of developing persistent cervical HPV infection in Filipino women.

## 1. Introduction

Cervical cancer is the second most common cancer site and the second most common cause of death due to cancer in the Philippines and globally [1]. There were 570,000 cases and 311,000 deaths worldwide from cervical cancer in 2018. More than 85% of these deaths were found in low- and middle-income countries. First-world countries such as the United States and China are also vulnerable to the high burden of HPV infection. As of 2021, the reported number of annual cervical cancer cases in the United States was about 13,545, while cervical cancer deaths amounted to 5706 cases [2]. Conversely, China had a higher number of annual cases, with 109, 741 cervical cancer cases and 59,060 cervical cancer deaths [3]. In the United States, the 5-year age-standardized relative survival rate for cervical carcinoma is 64.2% [4]. On the other hand, according to the Centers for Disease Control and Prevention (CDC), the 5-year survival rates for cervical cancer are about 58% for non-Hispanic Black women and 67% for non-Hispanic white women [5]. This is consistent with the previous study and was rationalized to be due to the diagnosis of an earlier stage of the disease.

Human papillomavirus infection is a necessary cause of cervical cancer [6] and is considered the most common sexually transmitted infection worldwide [7]. In the Philippines, cervical cancer is among the most common cancers, presenting an annual burden of 7829 new cases and 4052 deaths [1]. The estimated national standardized mortality rate for cervical cancer was 7.5 per 100,000. In total, 1 (0.7) out of 100 women would have died from cervical cancer before age 75. The five-year relative survival rate for Filipinos is 45.4% [8].

Numerous epidemiological studies in several countries have confirmed that human papillomavirus strongly correlates with cervical neoplasia. HPV DNA was detected in almost 93% of cervical cancer patients, with no significant variations in HPV positivity among countries [9]. A study from 1991 to 1993 in the Philippines showed that HPV DNA was present in 93.8% of patients diagnosed with squamous cell carcinoma and 90.9% of patients with adenocarcinoma/adenosquamous carcinoma. Fifteen different HPV types were detected in squamous cell carcinoma, while six HPV types were detected in adenocarcinoma/adenosquamous carcinoma [10].

One of the principal socioeconomic vulnerabilities cited for cervical cancer is poverty, thus explaining the lack of effective screening programs to detect and treat precancerous conditions and the inability to bear costs related to treatment, making cervical cancer incidence much higher in developing countries [9]. This includes other factors, such as system and practitioner delay, which are some healthcare-related challenges, and psychological factors, such as negative social attitudes and distress associated with treatments [11,12].

Human papillomavirus (HPV) infection is one of the most studied risk factors for cervical cancer [13]. Although HPV is essential in the development of oncogenicity, as it influences the conversion of cervical epithelial cells into their malignant counterparts, other factors and molecular occurrences are needed for cervical cancer to develop [14]. About 200 different HPV groups have been characterized, with the majority having low risk of developing cancer [15]. The individual viruses found within HPV groups are identified as high or low risk depending on their tendency to progress into malignant cells. Low-risk HPV viruses (e.g., HPV-6 and HPV-11) only produce localized benign warts and are non-malignant, while high-risk HPVs (e.g., HPV-16 and HPV-18) are linked to the development of high-grade cervical lesions and invasive carcinoma [16,17].

Of all high-risk HPVs, HPV-16 is the most prominent strain identified and associated with vaginal, vulval, anal, and penile cancers [18]. HPV-16 and HPV-18 are both known to be high-risk HPVs that have several functional coding regions that code for proteins that control the function of E6 and E7 genes [18,19]. E6 genes are known to inhibit negative regulators of the cell cycle and inhibit p53, a known transcription factor that activates apoptosis [18,20]. On the other hand, E7 codes for viral proteins that bind to retinoblastoma tumor suppressor proteins. Therefore, despite the absence of normal mitogenic signals, the cells progress in the cell cycle. In the Philippines, HPV-18 is the most common HPV type compared with other countries where HPV-16 is more prevalent [21].

Despite the risks, population-based epidemiological data on HPV in the general population in most parts of the Philippines remains unknown [22], which is emphasized by the low HPV coverage in the country [23]. There is limited knowledge of HPV prevalence and genotype distribution among healthy patients and those with cervical cancer. Based on our current knowledge, only one study has been conducted to determine polymorphisms and genetic variants that influence the development of cervical cancer [23,24]. However, this emphasizes the need for further studies in the field.

Determining the HPV genotype and distribution among the general population can help address the gap in the knowledge of the epidemiology of HPV in the Philippines [23,25]. The molecular epidemiology of HPV genotype distribution is essential to estimate the potential impact of prophylactic HPV vaccination and to assess the importance of HPV testing-based cervical screening [26,27]. This study will update the prevalence and genotypic distribution of HPV infection among Filipino women of reproductive age, which can determine whether available vaccines in the HPV vaccination program in the Philippines capture the most prevalent high-risk HPV genotypes in the country. In addition, this study will also identify vaginal community state types and bacterial taxa associated with different infection statuses of HPV. This can be used in developing a biomarker that can help predict the risk of developing persistent HPV infection and cervical cancer in Filipino women.

Papanicolaou smear testing leads to the early detection and treatment of precancerous cervical lesions in most developed countries with well-organized cytologic screening programs [28,29,30,31]. This prevents the progression of cervical lesions to cervical cancer [32,33]. However, in Asia, especially in developing countries, coverage of cervical cancer screening programs is still low, mainly because of limited access, lack of knowledge about the disease, and the absence of organized screening programs in some countries. These contribute to the high burden of cervical cancer in most Asian countries, including the Philippines [34].

Current screening strategies established in the country include pap smear testing and visual inspection with acetic acid (VIA) [35], which is the current recommended method by the Department of Health to be conducted at the population level, as it is more practical than Pap smear tests [36,37,38]. Studies have shown that in the past five years, the current screening coverage of HPV in the Philippines among women of reproductive age has been 2% [39], which is far from the 70% target coverage of the World Health Organization [40]. Numerous factors have been barriers to the current low-percent coverage of HPV screening programs in the country, including limited resources, geographic disparities, scarcity of workforce and funding, high out-of-pocket costs for screening, and the lack of knowledge of the importance of HPV screening programs [41]. There is a lack of population-based screening for cancers in the Philippines, and little information is available to quantify the delays in cancer interventions [42]. Most patients seek professional help in hospitals for initial evaluation or during severe cases that are difficult to treat, but most opt to look elsewhere for medical care, such as private medical service providers or traditional healers [43].

Multi-omics is a field of scientific research that integrates various omics data into one. This approach combines two or more omics data sets that are then analyzed, visualized, and interpreted to determine different biological processes based on a given scope [44]. Using a multi-omics approach, the study on HPV can be further integrated to guide future researchers in the novel identification of potential targets and biomarkers in preventing the development of cervical cancers and the progression of persistent HPV infections [45,46].

While it has already been established that HPV infection is a well-established risk factor for cervical cancer, some factors may contribute to cervical carcinogenesis [47]. Vaginal dysbiosis has been a long-standing theory on the development of cervical cancer [48]. Studies have shown that cervical microflora differs significantly in HPV-positive and HPV-negative women and in all stages of the natural history of cervical cancer [34,49,50]. Vaginal microbiomes were associated with the persistence and progression of high-risk HPV infection in a prospective longitudinal cohort [51,52]. Recently, a meta-analysis that included 15 prospective cohort studies showed a causal link between vaginal dysbiosis and cervical cancer [53]. However, these studies have not been conducted in the Philippines.

The present study protocol aims to determine the factors associated with the natural history of cervical HPV infection in a cohort of women in rural and urban Philippines centers using an omics approach. Specifically, it aims to (1) describe the sociodemographic profile and reproductive health behavior of the study cohort; (2) determine the cervical HPV infection prevalence and genotype distribution in the study cohort; (3) characterize the vaginal microbiome and metabolomic profiles of HPV-positive and HPV-negative study participants; and (4) identify factors influencing the acquisition, clearance, and persistence of cervical HPV infection in the study cohort after 6 and 12 months at follow-up examinations.

## 2. Materials and Methods

### 2.1. Study Design

**Figure 1:**
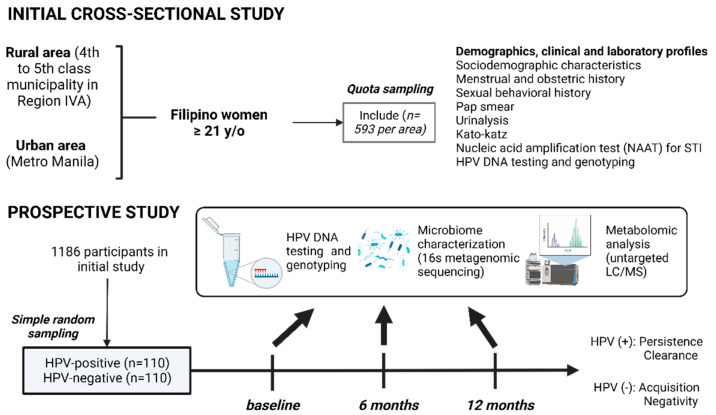
Schematic diagram of participant enrollment during the study and data collection at each time point in DEFEAT HPV study in the Philippines.

The DEFEAT HPV Study or the Determinants oF Acquisition, persistence, and clearance of oncogenic cervical Human Papillomavirus infection in a cohort of women in rural and urban Philippines using a multi-OMICs approach, will employ a community-based prospective cohort design. An initial cross-sectional study will identify age-matched HPV-positive and HPV-negative individuals, who will be invited to participate in the longitudinal study (Figure 1).

### 2.2. Sampling Design

The study will employ two non-probabilistic sampling techniques: convenience sampling for the selection of study sites and quota sampling for the recruitment of study participants. Patients will be recruited from the selected community centers and enrolled in the study if they satisfy the eligibility criteria. Recruitment and sample collection will continue until the desired number of participants is reached.

### 2.3. Sample Size

The sample size was based on Casals-Pascual et al., 2020, regarding the number of participants required to find significant differences in alpha diversity in clinical microbiome studies [54]. To find differences in microbial diversity between study groups with an effect size of 0.55 and adequate power (80%), at least 110 HPV-positive participants must be enrolled in the study. The most recent data from HPV and related cancers Fact sheet 2017 showed an HPV infection prevalence of 9.3% among women with normal cytology [55]. Using an estimated prevalence of 9.3%, precision of 5%, and 10% adjustment for nonparticipants, at least 1186 women (593 each in urban and rural centers) should be screened during the initial survey to identify 110 HPV-positive patients (http://sampsize.sourceforge.net/iface/index.html accessed on 1 January 2023) [56].

### 2.4. Study Sites

Recruitment of participants and subsequent sample collection will be performed in Likhaan Center for Women’s Health clinic in Tondo, Manila (urban site), and Naic, Cavite (rural site). These sites were selected based on good coordination between the principal investigator and the non-government organization, and the availability of trained local health personnel. Laboratory processing and molecular testing will be conducted at Virology Laboratory, Department of Medical Microbiology, College of Public Health, University of the Philippines Manila.

### 2.5. Study Population

#### 2.5.1. Inclusion Criteria

The eligibility criteria for the initial cross-sectional survey will be the following: women aged 21 years or older will be eligible to participate if they are currently sexually active or seeking birth control and have an intact uterus. The eligibility criteria for the longitudinal study will be the following: women aged 21 years or older included in the initial cross-sectional survey who underwent HPV DNA PCR and Pap smear tests.

#### 2.5.2. Exclusion Criteria

The exclusion criteria will be verified during the physician’s pelvic exam, by means of medical record, and/or self-reported. Written informed consent will be obtained from all participants. Exclusion criteria for the study will be current pregnancy; menstruating during sample collection; taking antibiotics, antifungals, or antivirals currently or within the previous three months; treatment for cervical intraepithelial neoplasia within the previous 18 months; having a current referral for hysterectomy or had a total or radical hysterectomy for any indication; use of douching substances, vaginal applied medications and suppositories, feminine deodorant sprays, or vaginal lubricants within 48 h prior the visit; any skin condition in the genital area interfering with the study; sexual intercourse less than 48 h prior the visit; and having type I or type II diabetes, hepatitis, and HIV infection.

### 2.6. Study Procedure

#### 2.6.1. Process for Securing Consent from the Participants

Informed consent will be given to target participants during the survey. They will be given enough time to decide whether to participate in the study. The research team will provide and discuss the research details, so they will be fully aware of the nature of the study. Advantages and potential disadvantages will be discussed in full disclosure with the participants. The research team will also provide the following information during the process of securing consent: (1) significance of the study; (2) detailed flow on the processes involved in the testing and validation of the samples they will submit; (3) disposal protocol of the samples they will submit; and (4) referral plan in case they will have abnormal examination findings.

#### 2.6.2. Referral Plan

Women receiving abnormal Pap smear results with positive PCR tests for oncogenic HPV will be referred to the rural health unit of their respective municipalities for appropriate management. These participants will be given follow-up diagnostic recommendations that range from repeat Pap smears at 6 and 12 months to colposcopy (with or without endocervical sampling) to loop electrosurgical excision.

#### 2.6.3. Grouping of HPV Status at One-Year Follow-Up

To investigate the shifts in bacterial communities in the HPV states, we will group the study participants based on the following conditions: (1) Group 1.1 (HPV negative)—women who tested persistently negative in the detection of HPV types throughout 12 months (all negative during the observation period); (2) Group 1.2 (HPV acquisition)—women who were HPV negative at baseline and then became HPV positive during the follow-up period; (3) Group 2.1 (HPV clearance)—women who were HPV positive at baseline and then became HPV negative during the follow-up period; (4) Group 2.2 (HPV persistence)—women who were persistently HPV positive at baseline and during the follow-up periods. Patients will be asked to follow up for the study two times, 6 and 12 months after the first visit.

### 2.7. Data Collection Tools

#### 2.7.1. Clinical Interview and Examination

An interviewer-administered questionnaire will be used in this study. The data collection tool will be pre-tested for content and face validity, and field interviewers will be trained before field deployment. The following data will be collected: (1) sociodemographic characteristics (age, place of residence, educational attainment, monthly family income, marital status, alcohol drinking, and smoking); (2) medical history (gravidity and parity, age of menarche, number of lifetime sexual partners, history of sexually transmitted infections, use of oral contraceptives, and previous Pap test or visual inspection with acetic acid); (3) awareness and practices of cervical cancer preventive behavior; (4) sexual and reproductive health behavior (use of contraceptives, number of children, age at first oral intercourse, lifetime oral sexual partners, age at first vaginal intercourse, and lifetime vaginal sexual partners). A licensed physician will conduct standard gynecological examinations. These will be performed in a private room with the assistance of a nurse.

#### 2.7.2. Sample Collection and Transport

A licensed physician will collect cervical and vaginal swabs. A sterilized speculum will be inserted without lubricant. The cervical and vaginal swabs will be collected by swabbing the cervix and the lateral walls of the mid vagina using an ENAT PM 2 mL L–shape Applicator (Cat. No. 608CS01L; Seegene Inc., Seoul, South Korea) and an ENAT PM 2 mL Regular Applicator (Cat. No. 608CS01R; Seegene Inc.), respectively. The samples will then be immediately placed on ice and frozen at −80 °C within 1 h of collection. All samples will be subjected to a Pap smear test. A licensed pathologist will conduct a cytology report according to the Bethesda system. The cytology report will be categorized into normal and abnormal. Routine cervical cytology will include negative for intraepithelial lesion or malignancy (NILM) and inflammatory changes. Abnormal cervical cytology will consist of atypical squamous cells of undetermined significance (ASC-US), low-grade squamous intraepithelial lesion (LSIL), high-grade squamous intraepithelial lesion (HSIL), and squamous cell carcinoma (SCC). Urine and stool samples will be collected and submitted for routine urinalysis and stool examination for the detection of parasitic infections. These tests will be performed by a licensed medical technologist and pathologist.

#### 2.7.3. Genomic DNA Extraction

Genomic DNA will be extracted from cervical and vaginal swabs using the Qiagen DNA Extraction Kit according to the manufacturer’s procedure. Extracted DNA in aqueous solution will be quantified (ratios of 260/280 and 260/230) using NanoDrop 2000 Spectrophotometer (Thermo Fisher Scientific, Wilmington, DE, USA). Sample adequacy will be evaluated by means of the amplification of the human β-actin gene using 1 μL of genomic DNA, 6.25 μL of 2x QuantiTect^®^ SYBR^®^ Green, 0.625 μL of each 10 μM primer, and 4 μL of deionized distilled water, in a total volume of 12.5 μL. PCR will be conducted with initial denaturation of 95 °C for 15 min, followed by 35 cycles of denaturation at 95 °C for 20 s, annealing at 56 °C for 1 min, extension at 72 °C for 1 min, and final extension of 72 °C for 4 min. All samples that present positive for β-actin will be used for the detection of HPV genotyping [57].

#### 2.7.4. HPV Genotyping

The extracted genomic DNA from the cervical swabs will be subjected to HPV genotyping using Anyplex II HPV28 Detection Kit (Cat. No. HP7S00X; Seegene Inc.) following the manufacturer’s instructions. This test can detect the HPV DNA of 28 anogenital HPV types [58,59].

#### 2.7.5. Detection of Lower Reproductive Tract Infections

The genomic DNA extracted from the vaginal swabs will be used to detect lower genital tract infections (Chlamydia trachomatis, Neisseria gonorrhoeae, Mycoplasma genitalium, Mycoplasma hominis, Ureaplasma urealyticum, Ureaplasma parvum, and Trichomonas vaginalis). AnyplexTM II STI-7 Detection (V1.1) (Cat. No.SD7700X, Seegene Inc.) will be used following the manufacturer’s instructions. This kit is a cost-effective and rapid diagnostic tool for detecting multiple sexually transmitted infections [60,61].

Bacterial vaginosis (BV) will be assessed using Amsel criteria [62] and the Nugent score [63]. A licensed medical technologist and physician will process and assess the samples. At least three of the four Amsel criteria will be required for diagnosing BV using Amsel criteria [64].

#### 2.7.6. 16S Amplicon Metagenomic Sequencing Using 454 GS-FLX Plus

The protocol for metagenomic sequencing will be adapted from a previous study on the association of the cervical microbial community and HPV infection status in Korean women [50]. The raw sequences for the samples will be arranged using a unique barcode, and low-quality reads (average quality score <25 or read length <300 bp) will be removed. The primer sequences will be cut down by employing pairwise sequence alignment, and sequences will be gathered to correct for sequencing errors. Taxonomic identification will be performed using the EzTaxon-e public database according to the highest pairwise similarity among the BLASTN search results. The UCHIME algorithm will remove possible chimera sequences, and the diversity indices will be calculated in Mothur after normalizing the read number in each sample. The potential biomarkers linked to HPV negativity, HPV clearance, and HPV persistence will be analyzed using Liner discriminant analysis (LDA) effect size (LEfSe). Finally, the effect relevance will be predicted using LDA.

#### 2.7.7. Metabolite Extraction and Separation Using LC/MS

Briefly, cervicovaginal samples (50 μL) will be extracted using a 500 μL methanol. The aqueous supernatant will be vacuum dried and reconstituted using water:acetonitrile (4:1, *v/v*) solution [65]. A QC sample will be prepared by pooling aliquots of 1uL from each sample. LC-MS will be conducted using a Vanquish UHPLC system in tandem with the Orbitrap Fusion Tribrid with a heated electrospray ionization source. A gradient elution using 0.1% FA in water (A) and 0.1% FA in acetonitrile (B) will be done using an Acclaim C18 column. A full scan MS analysis via the Orbitrap detector will first be employed on the QC sample. This will be injected after every five samples to serve as a reference. Data-dependent acquisition using dynamic exclusion at a scan range of 80–1000 will be subsequently performed on the individual samples via collision-induced dissociation (CID) with a linear ion trap detector.

The data will be analyzed using Compound Discoverer 3.2. The pre-set workflow ‘Untargeted Metabolomics with Statistics Detect Unknowns with Mapped Pathways and ID using Online Database’ was used as a framework for the analysis with a few modifications to fit the sample study. The data will be pre-processed using the software’s own algorithm, which includes retention time alignment and feature detection. Missing value imputation using Random Forest will be used for dealing with missing values. Molecular networks will be generated to create associations between spectra with high similarity. Metabolika Pathways will be used to determine the significant biochemical pathways involved. mzCloud search will be used as the main database for putative identification. Hits with no mzCloud results will be putatively identified using ChemSpider database search.

### 2.8. Quality Control

Licensed physicians will perform all gynecological examinations with training on the proper collection of cervical swabs for cytology. Two board-certified pathologists will independently examine the cytology samples. Data collectors and community health workers who will help in sample collection and interviews will be oriented and trained at the beginning of the study. English questionnaires will be translated into Filipino and backtranslated into English. A pre-test will be performed. Data will be encoded twice by two individuals and then checked by a third person.

### 2.9. Statistical Analyses

Descriptive analysis using tables, frequency (%), and mean values will be used to summarize and analyze the collected data. Descriptive analysis will be performed by obtaining quantitative variables’ means and standard deviations. Proportions and frequencies will be reported for qualitative variables. The chi-square test will be used to test the categorical variables, and the Kruskal–Wallis test will be employed to compare cohort characteristics based on HPV infection status. One-way analysis of variance with Tukey’s test will be used to assess differences in cohort characteristics based on the natural history of HPV infection, i.e., acquisition, clearance, or persistence of the infection.

The multivariable logistic analysis will be performed after adjusting for age, menopausal status, oral contraceptive use, and smoking habit. A logistic model with a robust variance will be estimated to compare those with HPV persistence to those with HPV clearance and HPV negativity. Hazard ratios (HRs) and 95% confidence intervals (CIs) will be obtained from the models as measures of association. The statistical analyses will be performed with SAS 9.4, and R version 3.3.1 with ggplot2 packages will be used for visualization. The results will be considered statistically significant if *p* < 0.05.

### 2.10. Ethical Consideration

This study was approved by the University of the Philippines Manila Research Ethics Board (UPMREB 2021-0645-01) on 25 January 2022. Informed consent will be obtained from the participants invited to participate in the study by one of the investigators or the research assistant. The informed consent form will be in both English and Filipino. If the patient is illiterate, the researcher/person taking consent will read out the informed consent form in the presence of a literate witness. Participation in the study will be voluntary, with the option to opt-out without reason and any penalty or issue. Assurance of privacy and confidentiality and declaration of risks, benefits, compensation, and conflicts of interest will be stated in the informed consent. The study will only proceed after the signing of the certificate of informed consent in the said form by the participants. All procedures performed in this study will follow the ethical standards of the institutional research committee and with the Helsinki Declaration and its later amendments.

## 3. Results

The primary outcomes of this study include the prevalence and genotype distribution of HPV infection among women in urban and rural communities in the Philippines. This study will also document the persistence or clearance of HPV infection among HPV-positive study participants and the incidence of HPV acquisition and negativity among initially HPV-negative participants after 6 and 12 months. This study will also determine the prevalence of reproductive tract infections (i.e., *C. trachomatis*, *N. gonorrhoeae*, *M. genitalium, U*. *parvum*., *U. urealyticum, T. vaginalis*, and bacterial vaginosis) among study participants.

To further understand the risk factors associated with HPV infection in the Philippines, the study will also determine the sexual and reproductive health behavior of participants. Associations of sociodemographic factors and health behaviors and the status of HPV infection will be obtained.

The vaginal microbiome and metabolomic profiles of patients will be obtained at the time of enrollment, after 6 months, and after 12 months. Associations of vaginal microbiome and metabolome and the status of HPV infection among study participants will be performed. Distinct microbiome and metabolomic signatures can be used as potential biomarkers for persistent HPV infection, a necessary cause of cervical cancer.

## 4. Discussion

Patients with cervical HPV infection do not commonly present symptoms. However, some patients with persistent infection may manifest anogenital warts [14]. Persistent infection with high-risk HPV may lead to the development of cervical cancer [23]. In the Philippines, women are at high risk of contracting the infection and developing the disease. The country ranked last in the HPV vaccination program coverage compared with other low–middle-income countries as of 2021 [66]. These data showed 23% and 5% program HPV vaccination coverage rates in women for their first and second doses, respectively [67].

Although cervical cancer is a preventable and manageable disease, various limitations in the Philippines put Filipino women at high risk of contracting and dying from HPV infection and cervical cancer. HPV vaccines were made available in 2006, in alignment with the thrusts of the World Health Organization (WHO) to immunize individuals to prevent the development of HPV and cervical cancer [68]. Currently, a global agenda is in place, as the WHO aims to eliminate HPV as a public health problem by 2030 through the vaccination of at least 90% of 15-year-old females, the screening of 70% of women aged 35 years old through high-performance tests and a follow-up screening test by age 45, and by providing management and treatment to preinvasive and invasive cervical cancer cases in 90% of the women identified [68,69]. However, it was only in 2015 that HPV vaccination was incorporated into the National Immunization Program (NIP) [69] of the Philippine’s Department of Health, which was followed by HPV-specific school-based vaccination in 2017 for school-age female children [70].

The lack of updated epidemiological information on HPV infection and cervical cancer in the country emphasizes the need for more research in this field. Despite recent efforts, morbidity and mortality from HPV infection and cervical cancer remain high [10,21,24]. Monitoring those at high risk of contracting HPV needs to be strengthened. The current HPV vaccination program needs to be assessed and evaluated based on its impacts on the current Filipino population [21,23]. The gains of the current project and its gaps must be determined to create a far more strengthened and effective system. The epidemiological data to be gathered in this study will provide evidence for strengthening health policies and programs on HPV vaccination and cervical cancer screening in the Philippines [21,42]. Data on the estimates of HPV infection and co-infections with other lower genital infections among urban and rural reproductive-age Filipino women will be generated. Not only will this study generate data for translation and interpretation by local health communities and fellow researchers, but the participants will also be given free Pap tests and HPV screening during the study as part of the expected outcomes of this research, particularly in its impact on and service to the community. The study reinforces the importance of reproductive health and preventive care with a primary care approach in the Philippines. Through the generated data, the study will provide insight into the extent of the HPV disease burden in the Philippines. It will, therefore, guide decision-makers in strategizing public health programs to control and eliminate HPV in the country. Moreover, the project results may have implications for the necessity of HPV vaccination among Filipino women of reproductive age.

The molecular assessment of the characteristics of HPV genotypes specific to the Philippines remains scarce. There is also limited information on the potential risk factors associated with persistent cervical HPV infection. It may require further validation to help determine the predisposed susceptibility of Filipino women to infection and the possible means of immunization that can be discovered [23]. Furthermore, assessing the various HPV genotypes requires routine assessment to strengthen treatment modalities. Type-specific HPV persistent infection may lead to failure in treatment due to the incomplete elimination of HPV infection, cervical intraepithelial neoplasia (CIN), and the likelihood of cervical cancer progression [23] This indicates the need for more frequent and consistent surveillance and the identification of individuals infected with HPV. Moreover, this study will also determine the vaginal microbiome, metabolome, and other sexually transmitted infections associated with persistent cervical HPV infection. These data may be used to develop diagnostic kits for persistent cervical HPV infection designed for the Filipino population. Moreover, prognostic markers using the microbiome and metabolome data may also be developed to help stratify patients at risk of having persistent cervical HPV infection and cervical cancer.

Only about 50–60% of patients diagnosed with cervical cancer receive some form of treatment [27], which emphasizes one of the difficulties faced by low–middle-income countries in defeating HPV. Treatment proves difficult, with 23.7% of Filipinos living below the poverty threshold [28] and 44.7% utilizing out-of-pocket expenses for healthcare services [8,71]. The management of late-stage cervical cancer entails high treatment costs in government hospitals. For every patient, multiple expensive imaging modalities (CT scan, MRI, and ultrasound) are needed in addition to the prices of chemotherapy regimens and surgical procedures. Hence, the disease burden also has implications on government expenditures, such that screening, prevention, and early intervention in HPV infection becomes imperative to decrease the economic burden of cervical cancer. This study will provide insights into the local molecular epidemiology of HPV infection so that public health strategies (e.g., prophylactic and screening measures, behavioral interventions, and vaccination) can be guided to effectively decrease the prevalence of HPV infection and HPV-associated cancers in the Philippines [26,72].

Other projects are also in place in the country to intensify the fight against HPV and cervical cancer, such as the Scale up Cervical Cancer Elimination with Secondary prevention Strategy (SUCCESS) project, which has been recently launched by the Department of Health in partnership with Jhpiego and funded by Unitaid on 12 April 2021 [73]. The project shows potential, as it aims to provide enhanced and simplified screening tests for HPV, capacity building, and preventive treatment through mass information dissemination. The DEFEAT HPV Project will complement the existing HPV and cervical cancer prevention and testing in the country and expand coverage.

### Limitations of the Study

Among the current limitations foreseen by the researchers in this project are its selection criteria, which include urban and rural areas in the Philippines. With this, the results of the study may not be generalizable to the whole country and can only account for a representative sample of the total population. Furthermore, the follow-up period of the study is only limited to 12 months.

Regardless of the limitations, the DEFEAT HPV study provides a novel perspective on assessing the prevalence of HPV among Filipino women at high risk of contracting HPV and thereby developing cervical cancer. This presents an excellent opportunity not only to improve HPV screening coverage in the country but also to upscale current efforts toward reaching the 2030 target of the World Health Organization to accelerate the elimination of cervical cancer as a public health problem (i.e., 70% of screening coverage; 90% of HPV vaccination; and 90% of treatment in women with pre-cancer and 90% in women with invasive cancer managed) [74].

## 5. Conclusions

In conclusion, the results of this study will be used to update the current prevalence and genotypic distribution of cervical HPV infection in the reproductive-age Filipino women population. With the use of a multi-omics approach, this study will improve our understanding of the epidemiology of cervical HPV infection in the Philippines. This is because multi-omics could provide researchers with a greater understanding of the flow of information regarding HPV, as it assesses the original cause of the disease (genetic, environmental, or developmental) to determine its functional consequences and interactions during development. This study can provide new information that can be used to guide public health programs and clinical practice guidelines on cervical HPV infection in the Philippines. 

## Data Availability

Not applicable.
